# Index Cohesive Force Analysis Reveals That the US Market Became Prone to Systemic Collapses Since 2002

**DOI:** 10.1371/journal.pone.0019378

**Published:** 2011-04-27

**Authors:** Dror Y. Kenett, Yoash Shapira, Asaf Madi, Sharron Bransburg-Zabary, Gitit Gur-Gershgoren, Eshel Ben-Jacob

**Affiliations:** 1 School of Physics and Astronomy, Tel-Aviv University, Tel-Aviv, Israel; 2 Faculty of Medicine, Tel-Aviv University, Tel-Aviv, Israel; 3 School of Business and Management, Ben Gurion University, Beer Sheva, Israel; 4 Department of Economic Research, Israel Securities Authority, Jerusalem, Israel; Universita' del Piemonte Orientale, Italy

## Abstract

**Background:**

The 2007–2009 financial crisis, and its fallout, has strongly emphasized the need to define new ways and measures to study and assess the stock market dynamics.

**Methodology/Principal Findings:**

The S&P500 dynamics during 4/1999–4/2010 is investigated in terms of the index cohesive force (ICF - the balance between the stock correlations and the partial correlations after subtraction of the index contribution), and the Eigenvalue entropy of the stock correlation matrices. We found a rapid market transition at the end of 2001 from a flexible state of low ICF into a stiff (nonflexible) state of high ICF that is prone to market systemic collapses. The stiff state is also marked by strong effect of the market index on the stock-stock correlations as well as bursts of high stock correlations reminiscence of epileptic brain activity.

**Conclusions/Significance:**

The market dynamical states, stability and transition between economic states was studies using new quantitative measures. Doing so shed new light on the origin and nature of the current crisis. The new approach is likely to be applicable to other classes of complex systems from gene networks to the human brain.

## Introduction

The current financial crisis began with the collapse of the subprime bubble at the end of 2007 [1], [2], and then spread to the global financial markets and economies worldwide. In the past, and in the aftermath of the crisis, much work has been devoted to the study and characterization of financial bubbles [1], [3], [4], [5], [6], [7], [8], [9]. In a recent study, Sornette *et al.* [1] have presented a general framework in which they propose that the fundamental cause of the crisis was in fact an accumulation of several bubbles in the markets, and the interplay between these bubbles.

The formation of bubbles in the markets is followed by a strong herding phenomenon amongst traders [9], and the burst of these bubbles is accompanied by strong synchrony in the markets reminiscent of epileptic seizures. For example, Lillo *et al.* [10], [11] have investigated the dynamics of markets following crashes. Such synchrony in the markets ca be used as a predictive measure for the formation of bubbles, and more importantly, for the burst of such bubbles. As such, it is crucial to develop new quantitative measures to fully capture, characterize and understand the market dynamical states, stability and transition between economic states. Currently, in this regard, much work is focused on the analysis of zero lagged [12] or higher-order lagged correlations [13], a detrneding approach to the study of cross correlations [14], [15], [16], and other measures to study co-movement and synchronization in stock markets [17], [18].

Here, we use a new, physics motivated, analysis framework to investigate the dynamics of markets, during the past decade. We show that the fragility of the market could be detected as early as the beginning of 2002, when the market dynamics went through a rapid change that was marked by a jump in the index cohesive force (ICF), and a decline in the correlation Eigenvalue entropy. This transition in the market dynamical state created a significant change in the structure of the market, due to an abnormal dominance of the market index on the stock correlations. The outcome was a rapid transition into a stiff market state that lacked a sufficient degree of freedom and internal flexibility of response to extreme changes. Hence, the index dominance rendered the market prone so systemic collapses as in the case of the sub-prime crisis.

We investigated the time dynamics of the S&P500 index, and 418 of its constituting stocks (not all 500 stocks were traded for the entire time period), during the last decade – from April 1999 to April 2010 (see also Text S1, for full description of the dataset). The investigations were carried out in terms of the index cohesive force (ICF) - the balance between the raw stock correlations that include the index effect and the residual stock correlations (or partial correlations) after subtraction of the index effect [19], [20]. The ICF provides a means to identify structural changes in the market, which significantly alter the stability of these markets. For additional assessments of the results we also inspected the time evolvement of the correlation entropy - the Eigenvalue entropy [19], [21], [22] of the matrices of stock correlations, during the last decade.

## Methods

### Raw Stock Correlations

The similarity between stock price changes is commonly calculated by the Pearson's correlation coefficient [20]. The raw stock correlations [20], [23] are calculated for time series of the log of the daily return, given by:
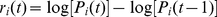
(1)Where 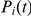 is the daily adjusted closing price of stock *i* at day *t*. The raw stock correlations are calculated using the Pearson's correlation coefficient *C*(*i,j*) between every pair of stocks *i* and *j*, where
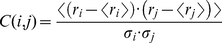
(2)

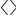
 denotes average, and 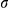 are the standard deviations (STD).

### Residual Correlations

Recently, we have made use of partial correlations to calculate the residual correlation between stocks, after removing the affect of the index [20]. Partial correlation is a powerful tool to investigate how the correlation between two stocks depend on the correlation of each of the stocks with a third mediating stock or with the index as is considered here. The residual, or partial, correlation 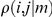 between stocks *i* and *j*, using the Index (*m*) as the mediating variable is defined by [19], [20], [24]

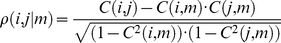
(3)Note that according to this definition, 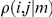, can be viewed as the residual correlation between stocks *i* and *j*, after subtraction of the contribution of the correlation between each of the stocks with the Index.

### The index cohesive force

Recently, we have shown that the market index has a cohesive effect on the dynamics of the stock correlations [20]. This refers to the observed affect the index has on stock correlations, where we have found that larger changes of the index result in higher stock correlations, and as such more cohesive [20]. Here we expand this analysis and introduce a quantitative measure of the index cohesive force. We define 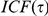 - the index cohesive force calculated over a time window 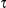, as a measure of the balance between the raw and residual correlations given by,
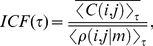
(4)where 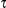 the time window, during which the average correlation and average residual correlation are calculated, denoted by 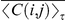 
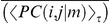
 is the average of average correlation (partial correlation). The size of the time window is selected following the considerations presented further below and in the Text S2 (see also Figure S7).

### Eigenvalue entropy

To further asses the market stiffness, we computed the eigenvalue (spectral) entropy of the raw correlation matrices. Qualitatively, the entropy of a system refers to the changes in the status quo of the system, and is used as a measure for the order and information content of the system. The spectral entropy [19], [21], [22], [25], [26], SE, is defined as
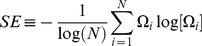
(5)where 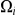 - the normalized eigenvalues 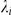 of the diagonalized matrix (correlation matrix) - are defined as
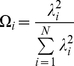
(6)Note that the 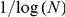 normalization was incorporated to ensure that SE = 1 for the maximum entropy limit of flat spectra (equal eigenvalues). We associate the market stiffness with one minus the SE [19], [21], [22], [25], [26].

## Results

The average raw correlation between stocks has been investigated in the past [27], [28], [29], [30], with the focus being on large time windows (200 to 500 days) to reduce the statistical variations. Here we selected a shorter, 22 trading days (corresponding to one work month), time window. We validated that while these short time windows retained limited variations (as shown by the results), they are successful in capturing short time events in the market dynamics. Such short time localized events are averaged out and cannot be deciphered when long time windows are used. In particular, we will show that using these short time windows enabled us to reveal changes in the index cohesive force that are very rapid and of high magnitude (see also Text S2 and Figure S7).

### Time dynamics of the raw and residual correlations and market stiffness

**Figure 1:**
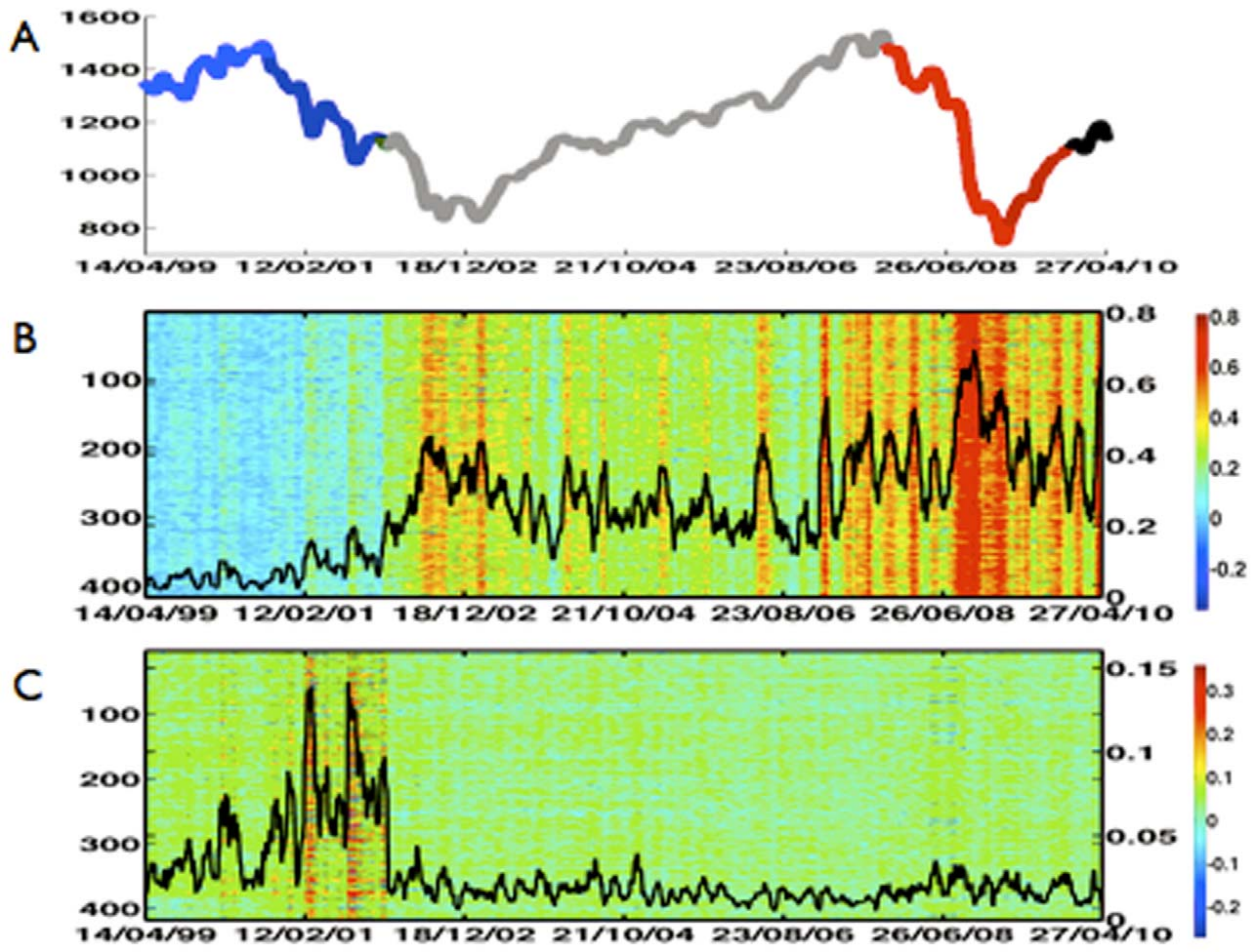
The dynamics of correlations and partial correlations for stocks belonging to the S&P500 Index from April 1999 till April 2010, versus the price of the Index. **A**) The S&P500 market index from April 1999 until the end of April 2010 Different time periods are marked by different colors: blue – April 1999 to December 2001, green – December 2001 to January 2002 (transition period), grey – January 2002 to July 2007, light red – August 2007–March 2009 (crisis), red – March 2009–January 2010 (recovery), and black – January 2010 to April 2010; **B**) Raster plot of the stock raw correlations, calculated according to the stocks daily returns and for 22 trading days windows. Each row shows the averaged correlations of a specific stock with all other stocks (left y axis), with the mean stock raw correlations (over all the stock correlations) superimposed in black (right y axis). **C**) Raster plot of the stock residual correlations after subtracting the index contribution, with the mean market residual correlations superimposed in black. In panel A the different colors indicate different time periods. In panels B and C the colors of the raster plots represent the strength of the correlations, as indicated in the color bars at the right side of each plot.

We begin our investigation by studying the dynamics of the stocks' raw correlations (Figure 1B) and residual correlations (Figure 1C), in comparison to the dynamics of the S&P500 index (Figure 1A). Such analysis reveals a transition in the market, taking place at the end of 2001. Following the transition, the market entered into a state dominated by the index as is reflected by the very small residual correlations in the new dynamical state. This state is characterized by an abnormal dominance of the market index, and a state in which the effect other processes such as the influence of different economic sectors is drastically reduced. We propose, in light of the recent global financial events, that the outcome is that the strong index influence rendered the market into a stiff state that is less adaptable to financial changes and therefore is more prone to crises. In other words, being a complex system [20], [31], when the average interactions between the market stocks becomes very large, the market becomes inflexible and more sensitive to external changes and thus more prone to crises (see Text S1 and Figures S1, S2, S3, S4, S5 for validation tests of the results, and Text S3 and Figures S8, S9, S10 for error estimation of the measures used to quantify the dynamics of correlations).

### Market seizure-like behavior

The anomalous dominance of the index and the market dangerous stiffness of this market state since the end of 2001, is manifested by the emergence of market seizure-like behavior - bursts of very high stock raw correlations that usually coincide with local minima in the index (Figure 1B). Performing our analysis using longer time windows resulted in qualitatively similar results, in which the transition in the market was still captured, while the localized bursts of correlation were no longer present.

### Dynamics of the index cohesive force

**Figure 2:**
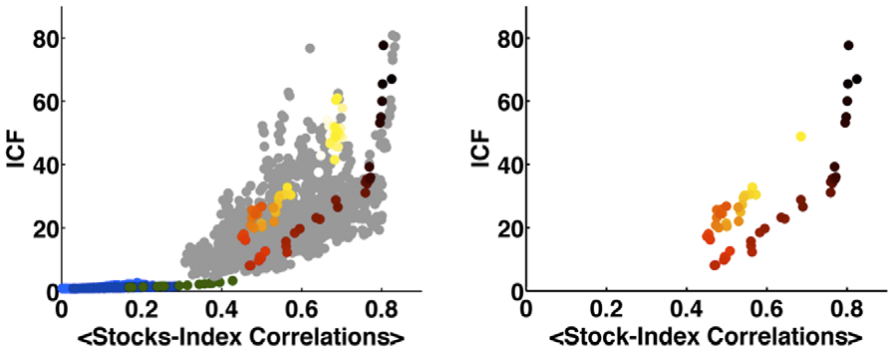
Time evolvement of the S&P500 market index cohesive force (ICF – the ratio between the raw correlations and the bare (partial) correlations), as function of the stocks-index correlations, during the last decade. The color code on the left is as in Figure 1A in the text. On the right, we only present the time progression during 2010, colored from light yellow at the beginning of the year to black at the end of April. Using this color code, we observe that during early 2010 the market dynamics moved back towards the stable state, but this trend was reversed at the end of March and currently the market instability seems to rapidly evolve towards a more fragile state.

In Figure 2 we present the time evolvement of the ICF, versus the average stocks-index correlations. In the left panel we use the same coloring scheme as in Figure 1A. The results well depict the significant difference between the two market states. In the right panel of Figure 2, we highlight the time period of 2010, using a color scheme from light yellow at the beginning of the year to black at the end of April. Using this color code, we observe that during early 2010 the market dynamics moved back towards the stable state, but this trend was reversed at the end of March (see also Text S1 and Figure S1).

**Figure 3:**
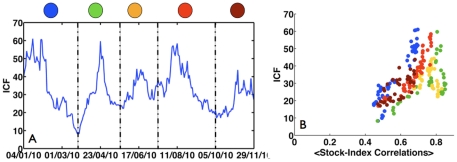
Time evolvement of the ICF for the entire year of 2010. (A) The ICF as a function of time, for 2010. We observe that the fluctuations of the ICF during 2010 were strong; we identify 5 different periods, which are characterized by changes in trend of the ICF. The Transition from the first period (blue circle) to the second (green circle) is similar to the one presented in Figure 2B. Furthermore, we observe two more strong peaks in the ICF – at June and in August of 2010. (B) Comparison of the ICF to the average stock index correlation, as presented in Figure 2, for the entire year of 2010. Color code as is indicated in Figure 3A.

To further assess the current state of the market, we calculated the ICF for the entire year of 2010. In Figure 3A we present the time evolvement of the ICF for 2010. We divide the entire year into 5 periods, based on the changes in the ICF. As was observed in Figure 2B, we find a drop in the ICF at the beginning of 2010 (blue circle), followed by a dramatic jump in the ICF (green circle). In addition to the strong peak in the ICF observed for April 2010, we observe additional somewhat weaker peaks, in June and August of 2010. Finally, as presented in Figure 2B, we compare the ICF to the average stock-index correlation, for the entire year of 2010 (Figure 3B, color coded according to Figure 3A). We note that in general, the year of 2010 was dominated by high values of the ICF, which remains high at the end of the year. Furthermore, comparing Figure 3B to Figure 2A, we observe that the market is still in the abnormal stiff state so it continues to be prone to systemic collapses.

### Reflections on the widely used systemic risk parameter

**Figure 4:**
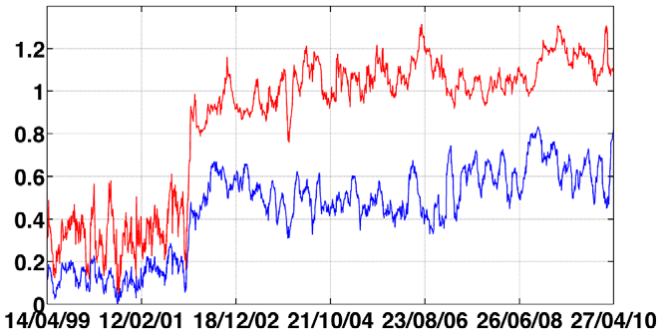
comparison of the average *β* (red curve) to the average stock-index correlation (blue curve).

In finance, the capital asset pricing model (CAPM) [32], [33] is used to determine a theoretically appropriate required rate of return of an asset, if that asset is to be added to an already well-diversified portfolio, given that asset's non-diversifiable risk. The model takes into account the asset's sensitivity to non-diversifiable risk (also known as systematic risk or market risk), often represented by the systemic risk parameter beta (*β*) in the financial industry, as well as the expected return of the market and the expected return of a theoretical risk-free asset. The correlation *C*(*i,m*) between the return of the given stock *i* and the daily return of the market index *r_m_*(*t*), is similar to *β_i_* - the systematic risk parameter of this stock which is defined within the security characteristic line (SCL) theory [32], [34], [35]. More specifically, using these parameters, the return of the asset on the return of the index is given by,
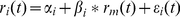
(7)Where 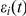 is a random variable and the regression parameters 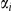 and 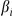 are given by:
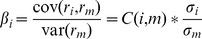
(8)

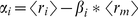
(9)According to these definitions, the residual correlation 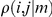 can be viewed as the correlation between the residuals 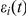, after removing the dependency of the given stock on the index. In Figure 4 we show that the average of the systematic risk 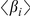 over all stocks (blue curve) differs from the average of the stock-index correlations 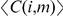 (red curve). As in the case of the average correlation, we observe a jump in 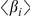 at the beginning of 2002. However, we did not decipher a trend reverse in the value of 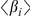 as we found for the ICF during the first months of 2010. We note that in a market which behaves as described by the Capital Asset Pricing Model (CAPM) [36], the 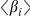 of the market should equal 1. In such market, as a result of its definition, the ICF should diverge. Hence, our results might indicate that the market dynamics do not follow the CAPM.

**Figure 5:**
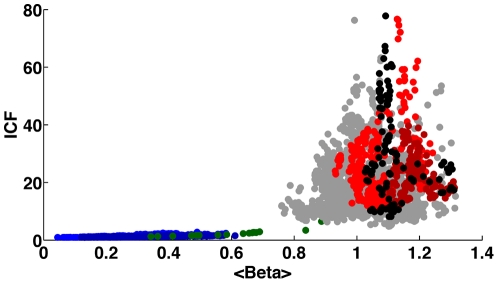
Comparison of the ICF to the 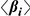 as function of time, color coded according to Figure 1A.

Furthermore, we present in Figure 5 a comparison of the ICF to the 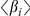 as a function of time, color-coded according to Figure 1A. It is evident that the two parameters are very different, especially following the transition at the end of 2001.

### Dynamics of Eigenvalue entropy

**Figure 6:**
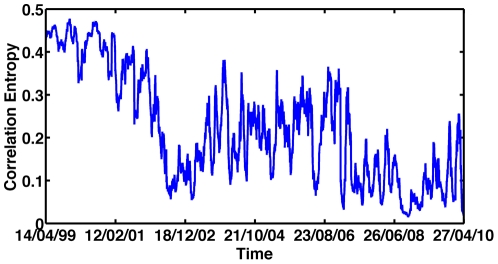
Correlation Eigenvalue entropy as function of time.

**Figure 7:**
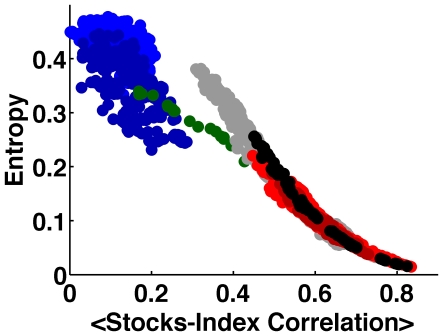
Eigenvalue entropy versus the average stock-index correlation, as function of time, color coded according to Figure 1A.

In Figure 6 we show the evolvement of the spectral entropy during the last decade. We note a sharp fall in the correlation entropy at the end of 2001, followed by strong entropy fluctuations. A second significant entropy fall is detected at September 2008 when the index dynamics switched from a negative trend to a positive trend. In Figure 7 we present the values of the entropy versus the average stock-index correlation, color-coded according to Figure 1A. This representation provides additional support that the market underwent a rapid transition between two very different dynamical states.

In a previous paper [19], we have studied how the entropy (information) content of a stock correlation matrix changes, when the market mode is removed. Either analyzing the partial correlation matrix, or looking at the eigenvalue spectrum without the principal eigenvalue can achieve this. Preliminary results (not shown) reveal that removing the principal eigenvalue dramatically influences the spectral entropy; while this is consistent with the rationale, the results are still inconclusive in this regard, and further research is necessary.

### Manifestation of the transition at the end of 2001

**Figure 8:**
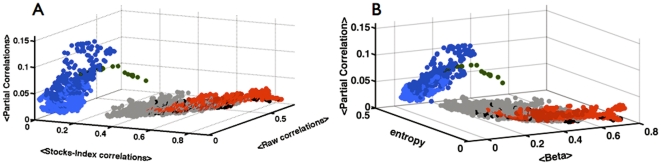
A three-dimensional scatter plot of the market dynamical evolution of stocks belonging to the S&P500 index in the past decade. (A) The axes are the average stocks-index correlations (X-axis), average raw stock correlations (Y-axis), and the average residual (partial) correlations (Z-axis). Each dot corresponds to a time window of 22 trading days and the color code is similar to that used in Figure 1A. (B) similar results are obtained when using longer time window and when replacing the average stocks-index correlation with the average *β* coefficient as the X-axis, and replacing the average stock-stock correlation with the entropy as the Y-axis.

The dramatic differences between the flexible and stiff (inflexible) market states are best manifested in the 3-dimensional scatter plot presented in Figure 8A. The axes of this 3D space are the average Stocks-Index correlations, the average raw correlations, and the average residual correlations. The color code makes transparent the fact that the market dynamical state was not determined by the Index trend (positive or negative): The stiff state started in the midst of a decline in the Index and continued unchanged as the Index trend changed several times. To demonstrate this change, we show in Figure 8B a scatter plot in a different 3D space – the axes are the spectral entropy SE, the average beta coefficient, 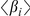 and the average residual correlations. Clearly the two scatter plots capture the same phenomenon. We also note that repeating the analysis while using the financial sector Index instead of the S&P500 Index yielded similar results.

## Discussion

In summary, we presented new approaches to quantify the dynamics of the stock market, using the correlation entropy and the index cohesive force (ICF). The ICF parameter provides a new quantitative measure to investigate different financial states of the market, and the transitions between these states.

Using this approach we discovered a rapid transition in the market dynamical state at the end of 2001. This transition is manifested by a jump in the stock correlations, and a sharp fall in the stock residual correlations. After the transition the market entered into a high ICF stiff state. In this state the index predominantly affects the market dynamics while it shades the effect of other degrees of freedom that can contribute to the market flexibility. Thus, we suggest that during this state the market is highly prone to systematic collapses, even due to relatively small external perturbations, leaving it incapable of coping with crises. This interpretation is consistent with the fact that following the burst of the subprime bubble and the fall of Lehman Brothers [37], [38], the market collapsed. It is also reasonable to assume that this rapid transition at the end of 2001 might have been a consequence of the “dot-com” bubble crisis, combined with the traumatic events which took place in the U.S. at the beginning of the decade and the outcome of the rapid interest cuts [39] and other financial policies employed to overcome the fallout effect of those. One such important financial policy was the implementation of the Decimal Pricing system in the American stock markets. The process of implementation was finalized in the NYSE at January 2001, and in the NASDAQ at April 2001. However, the observed transition in the market uncovered by the ICF took place at December 2001; thus, this change in tick size is one more contributing factor to the transition in the market.

The time period studied here covers the two largest crises that took place in the past decade – the 2000–2001 “.com” crisis, and the 2007–2009 credit crunch crises. During the “.com” period, internet and technological companies were hit hard by the crisis, while other sectors were less affected. This was a local crisis, and the bubble-crash was unevenly distributed among these sectors. This means that the residual correlations during this period should be unusually high, as indeed we found. The credit crunch crisis was a systemic (global) one, which spilled over from the financial sector into all other sectors. As such, the entire market dynamics exhibited high synchrony, as is reflected by the high values of the ICF measure introduced here. As we have shown, during the first part of 2010 there seemed to be a recovery in the markets, which was accompanied by a drop in the values of the ICF. However, a jump in the ICF, and indeed a renewed dangerous process in the market followed this drop in late March. Extending the analysis of the ICF to the entire year of 2010, we find that the ICF remains high; furthermore, short periods of relaxation in the ICF are followed by strong jumps in the ICF. Finally, we find that the end of 2010 is marked by an upwards trend in the ICF, which shows that the market is still in the abnormal state, and still strongly prone to systematic collapse.

Comparing between the ICF and the risk parameter *β*, we found that the ICF provides better representation of the state of the market: While *β* represents the coupling of a given stock to the index, the ICF represents the full state of the system, and can be considered as a system level measure of the state of the market. Probably for this reason, while the ICF revealed that during the first three months of 2010 the market was on its way to recovery, and then the trend was drastically changed at the end of March back into stiff state, this phenomenon is not revealed by the *β* parameter. Finally, the ICF parameter presented here can be further generalized, such as by a normalization of the volatility, or standard deviation of correlations; we propose this normalized ICF parameter as an Herding factor, which allows a quantification of herding in financial markets. A brief example of this Herd factor is presented in Text S1 (see Figure S6), and we plan on presenting a thorough investigation of this issue in the future.

In conclusion, we propose the ICF as a new system-level parameter, which provides an efficient measure to describe and quantify the market dynamical state, and which can be used as a tool to monitor the stability of stock markets. The stability of the markets is crucial for the world's economies, thus this tool can be very important to governments and regulation agencies worldwide.

## Supporting Information

Figure S1 Comparison of the ICF to the average stock-index correlation, for the period of 2010. The ICF and average correlation were computed for the 500 S&P500 stocks (left) and the 418 S&P500 stocks used for the entire analysis. We use a color code to present the chronological time progression, from dark blue for the beginning of 2010, to dark red, for April 2010. Comparing the two panels, we note that there is a high qualitative similarity between the two.
(TIFF)

Figure S2 Calculation of the ICF for a sub-set of 300 stocks. To validate the results of the ICF for the full dataset, we randomly chose 300 stocks, calculate the average stock, stock-index, and partial correlation, and the ICF. We perform this selection 4 times. The values of the ICF is presented for each of the 4 iterations, using a different color.
(TIF)

Figure S3 A three-dimensional scatter plot of the market dynamical evolution of stocks belonging to the S&P500 index in the past decade, as presented in Figure 8. We first calculate the average value of the raw, stock-index and partial correlations, over the 4 iterations of random selection of the 300 stock sub-set. The color code used is the same as in Figure 8.
(TIF)

Figure S4 Eigenvalue entropy versus the average stock-index correlation, as function of time, color coded according to Figure 1A. This is presented for the 300 stock subset, as in Figure S1, S2, S3. We first calculate the average value of the entropy and the stock-index correlation over all 4 iterations.
(TIF)

Figure S5 Comparison of the ICF calculated using the S&P500 index (red curve) and the ICF calculated using a synthetic index. The synthetic index was calculated using only the stocks included in the dataset, as a weighted average of these stocks, using their original weights from the S&P500 index. While the ICF calculated using the synthetic index is nosier, the two are qualitatively very similar, with a correlation of 0.65, which is probably strongly affected by the fact that the ICF(synthetic) is much nosier in the pre-2002 period.
(TIF)

Figure S6 Comparison of the H factor to the ICF, color coded for time according to the code presented in Figure 1A.
(TIF)

Figure S7 ICF analysis of the S&P500 dataset, using a sliding window of 50, 100, 200, 300, 400, and 500 days. The transition, observed using the 22-day window, is qualitatively observed for all other window sizes, around the same period.
(TIFF)

Figure S8 The average stock correlation (left) and average stock partial correlation (right), as resented in Figure 1 A and B respectively, with the addition of error lines. The error lines were estimated using the standard deviation for each parameter separately, marked by a dotted red line.
(TIFF)

Figure S9 The average Beta coefficient, as presented in Figure 4, with the addition of error, estimated using the standard deviation, marked with a dotted red line.
(TIF)

Figure S10 The value of the ICF as a function of time, with the addition of error markers, estimated by the standard deviations of the average correlation and average partial correlation, and the functional relation between them. The error boundaries are marked by a dotted red line.
(TIF)

Text S1 (DOC)

Text S2 (DOC)

Text S3 (DOC)

## References

[1] Sornette D, Woodard R (2010). Financial Bubbles, Real Estate Bubbles, Derivative Bubbles, and the Financial and Economic Crisis.. Econophysics Approaches to Large-Scale Business Data and Financial Crisis.

[2] Boyd JH, Jagannathan R, Kwak S (2010). What Caused the Current Financial Mess and What Can We Do about It?(Digest Summary).. CFA Digest.

[3] Bastiaensen K, Cauwels P, Sornette D, Woodard R, Zhou W (2009). The Chinese equity bubble: Ready to burst..

[4] Jiang Z, Zhou W, Sornette D, Woodard R, Bastiaensen K (2010). Bubble Diagnosis and Prediction of the 2005–2007 and 2008–2009 Chinese stock market bubbles.. Journal of Economic Behavior & Organization.

[5] Krawiecki A, Holyst JA (2003). Stochastic resonance as a model for financial market crashes and bubbles.. Physica A: Statistical Mechanics and its Applications.

[6] Sornette D, Woodard R, Zhou W-X (2009). The 2006–2008 oil bubble: Evidence of speculation, and prediction.. Physica A: Statistical Mechanics and its Applications.

[7] Kaizoji T, Sornette D (2010). Market bubbles and crashes..

[8] Woodard R, Sornette D, Fedorovsky M (2010). The Financial Bubble Experiment: Advanced Diagnostics and Forecasts of Bubble Terminations, Volume III..

[9] Lux T (1995). Herd behaviour, bubbles and crashes.. The Economic Journal.

[10] Lillo F, Bonanno G, Mantegna RN (2002). Variety of stock returns in normal and extreme market days: The August 1998 crisis.. Empirical Science of Financial Fluctuations.

[11] Lillo F, Mantegna RN (2004). Dynamics of a financial market index after a crash.. Physica A: Statistical Mechanics and its Applications.

[12] Plerou V, Gopikrishnan P, Rosenow B, Amaral LAN, Guhr T (2002). Random matrix approach to cross correlations in financial data.. Physical Review E.

[13] Podobnik B, Wang D, Horvatic D, Grosse I, Stanley H (2010). Time-lag cross-correlations in collective phenomena.. EPL (Europhysics Letters).

[14] Podobnik B, Horvatic D, Petersen AM, Stanley HE (2009). Cross-correlations between volume change and price change.. Proceedings of the National Academy of Sciences.

[15] Podobnik B, Stanley HE (2008). Detrended cross-correlation analysis: A new method for analyzing two nonstationary time series.. Physical Review Letters.

[16] Xu L, Ivanov PC, Hu K, Chen Z, Carbone A (2005). Quantifying signals with power-law correlations: a comparative study of detrending and moving average techniques.. Phys Rev E.

[17] Reigneron PA, Allez R, Bouchaud JP (2010). Principal Regression Analysis and the index leverage effect.. Arxiv preprint arXiv.

[18] Harmon D, de Aguiar MAM, Chinellato DD, Braha D, Epstein IR (2011). Predicting economic market crises using measures of collective panic.. Arxiv preprint arXiv.

[19] Kenett DY, Shapira Y, Ben-Jacob E (2009). RMT assessments of market latent information embedded in the stocks' raw, normalized, and partial correlations.. Hindawi Journal of Probability and Statistics.

[20] Shapira Y, Kenett DY, Ben-Jacob E (2009). The Index cohesive effect on stock market correlations.. The European Physical Journal B.

[21] Varshavsky R, Gottlieb A, Horn D, Linial M (2007). Unsupervised feature selection under perturbations: meeting the challenges of biological data.. Bioinformatics.

[22] Varshavsky R, Gottlieb A, Linial M, Horn D (2006). Novel unsupervised feature filtering of biological data.. Bioinformatics.

[23] Mantegna RN, Stanley HE (2000). An Introduction to Econophysics: Correlation and Complexity in Finance.

[24] Baba K, Shibata R, Sibuya M (2004). Partial correlation and conditional correlation as measures of conditional independence.. Australian & New Zealand Journal of Statistics.

[25] Alter O, Brown P, Botstein D (2000). Singular value decomposition for genome-wide expression data processing and modeling.. PNAS.

[26] Kenett DY, Shapira Y, Madi A, Bransburg-Zabary S, Gur-Gershgoren G (2010). Dynamics of stock market correlations.. AUCO Czech Economic Review.

[27] Laloux L, Cizeau P, Bouchaud JP, Potters M (1999). Noise dressing of financial correlation matrices.. Physical Review Letters.

[28] Bouchaud JP, Potters M (2003).

[29] Onnela JP, Chakraborti A, Kaski K, Kertesz J (2002). Dynamic asset trees and portfolio analysis.. European Physical Journal B.

[30] Onnela JP, Chakraborti A, Kaski K, Kertesz J (2003). Dynamic asset trees and Black Monday.. Physica A.

[31] Mauboussin MJ (2005). Revisiting market efficiency: the stock market as a Complex Adaptive System.. Journal of Applied Corporate Finance.

[32] Lintner J (1965). The Valuation of Risk Assets and the Selection of Risky Investments in Stock Portfolios and Capital Budgets.. Review of Economics and Statistics.

[33] Sharpe WF (1964). Capital asset prices: A theory of market equilibrium under conditions of risk.. Journal of Finance.

[34] Malevergne Y, Sornette D (2007). Self-Consistent Asset Pricing Models.. Physica A.

[35] Malevergne Y, Santa-Clara P, Sornette D (2009). Professor Zipf Goes to Wall Street..

[36] Black F, Jensen MichaelC,  Scholes Myron, Jensen M (1972). The Capital Asset Pricing Model: Some Empirical Tests.. Studies in the Theory of Capital Markets.

[37] Demyanyk Y, Hemert OV (2009).

[38] Sieczk P, Sornette D, Holyst JA (2010). The Lehman Brothers Effect and Bankruptcy Cascades..

[39] Taylor J (2009). The financial crisis and the policy responses: An empirical analysis of what went wrong..

