# Supplementary figures and images for: Index Cohesive Force Analysis Reveals That the US Market Became Prone to Systemic Collapses Since 2002
Source: PLoS One. 2011 Apr 27;6(4):e19378. doi: 10.1371/journal.pone.0019378 (PMC3083438; doi:10.1371/journal.pone.0019378)

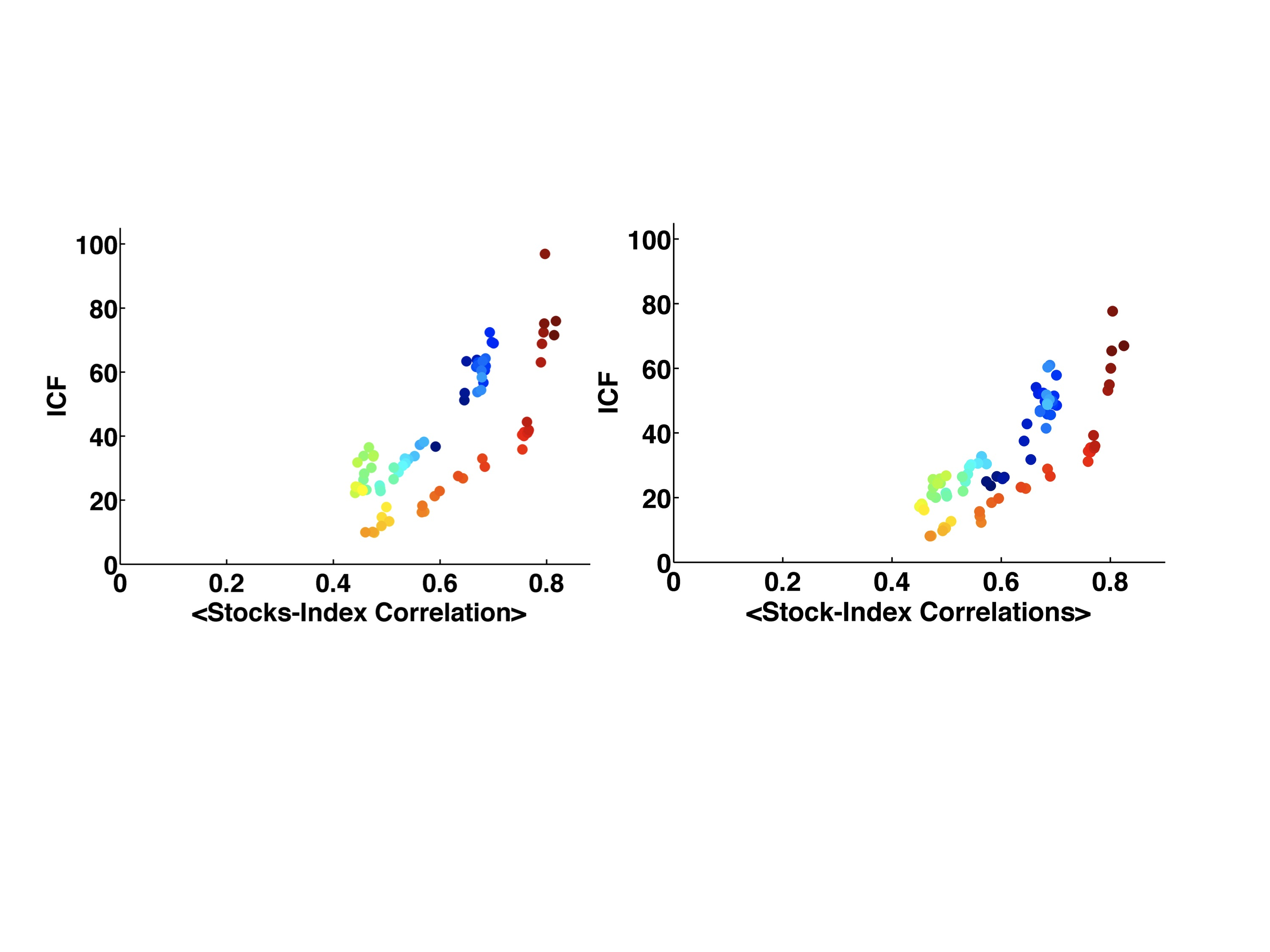

Supplement: Figure S1 — Comparison of the ICF to the average stock-index correlation, for the period of 2010. The ICF and average correlation were computed for the 500 S&P500 stocks (left) and the 418 S&P500 stocks used for the entire analysis. We use a color code to present the chronological time progression, from dark blue for the beginning of 2010, to dark red, for April 2010. Comparing the two panels, we note that there is a high qualitative similarity between the two. (TIFF) [file pone.0019378.s001.tiff]

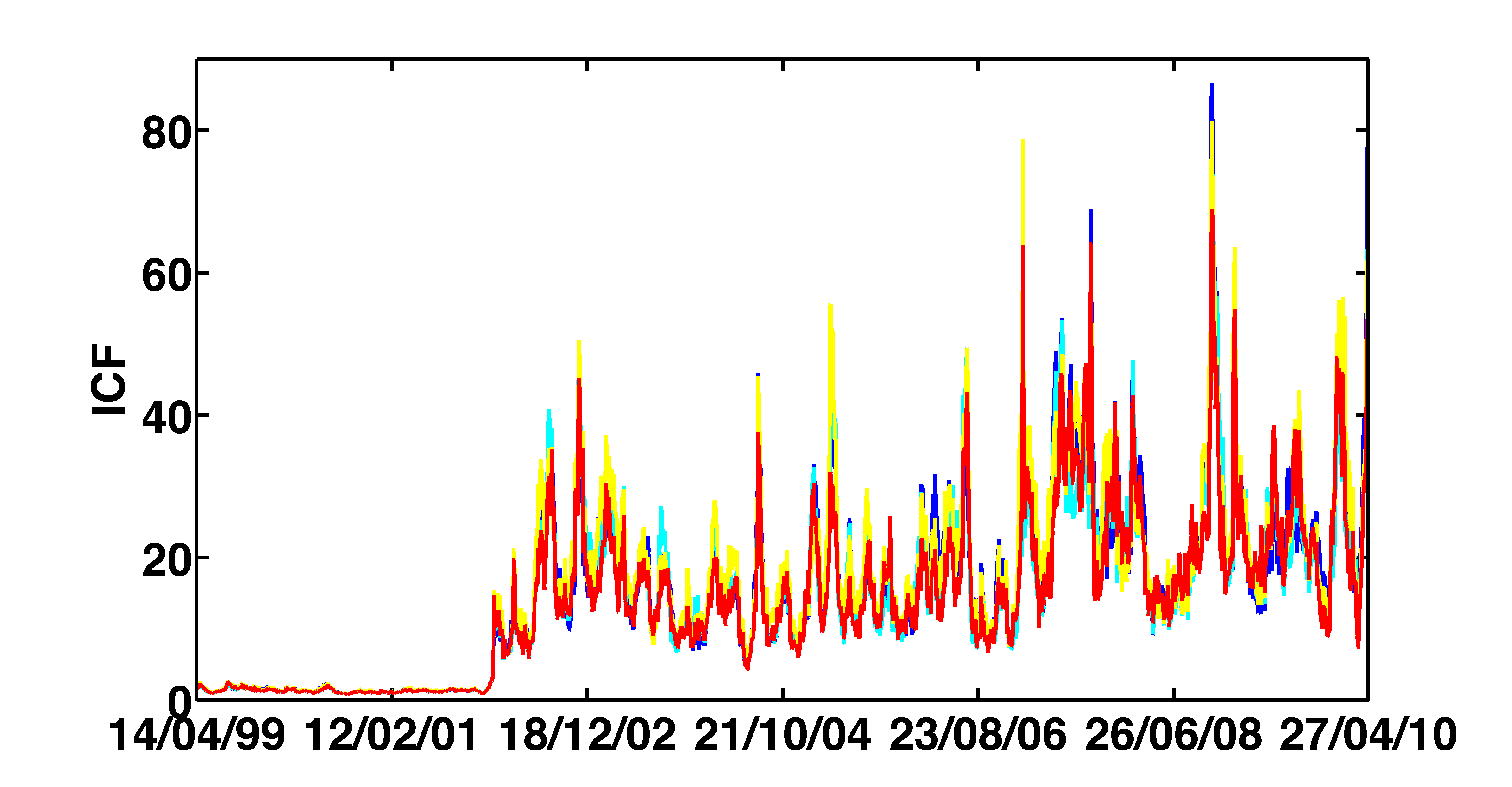

Supplement: Figure S2 — Calculation of the ICF for a sub-set of 300 stocks. To validate the results of the ICF for the full dataset, we randomly chose 300 stocks, calculate the average stock, stock-index, and partial correlation, and the ICF. We perform this selection 4 times. The values of the ICF is presented for each of the 4 iterations, using a different color. (TIF) [file pone.0019378.s002.tif]

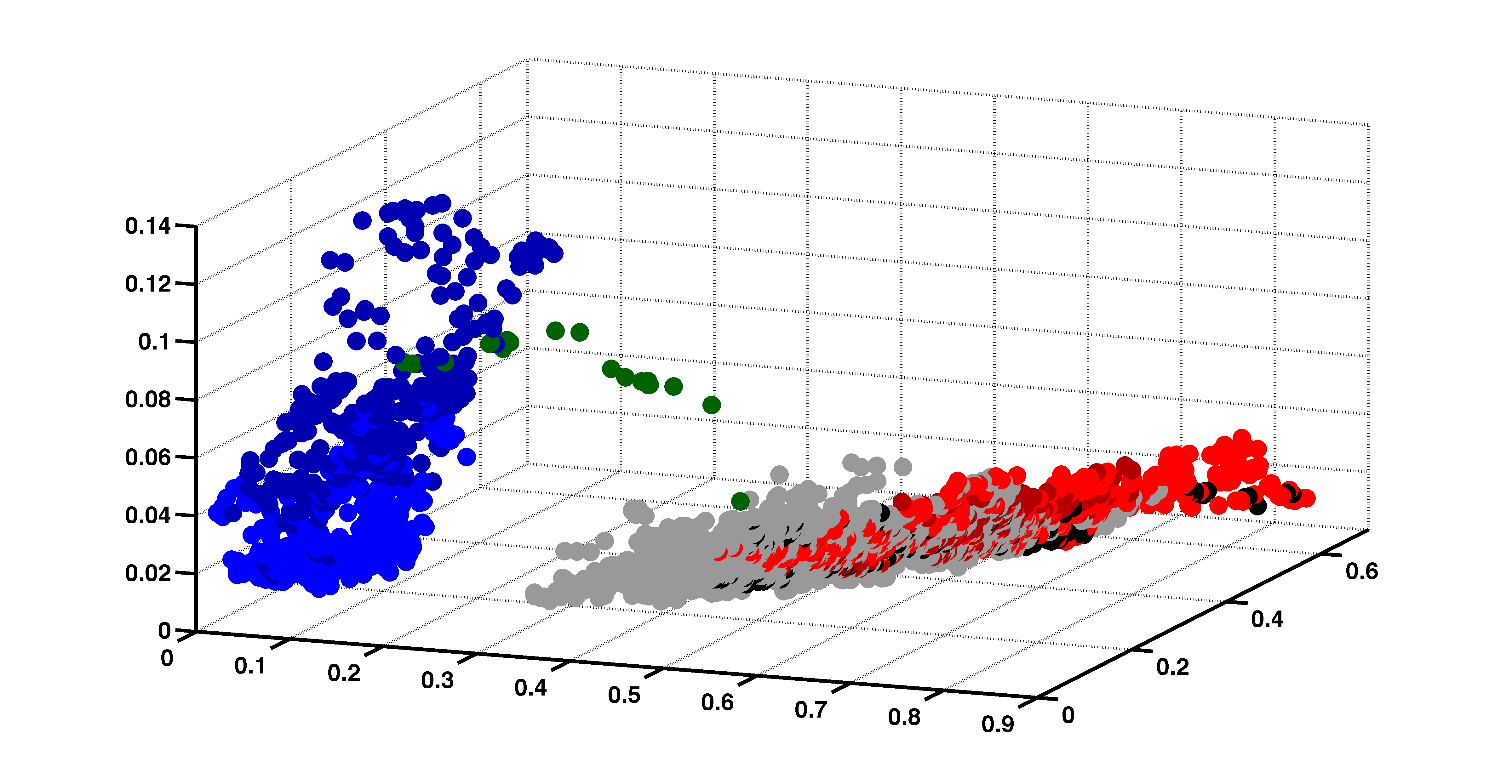

Supplement: Figure S3 — A three-dimensional scatter plot of the market dynamical evolution of stocks belonging to the S&P500 index in the past decade, as presented in Figure 8. We first calculate the average value of the raw, stock-index and partial correlations, over the 4 iterations of random selection of the 300 stock sub-set. The color code used is the same as in Figure 8. (TIF) [file pone.0019378.s003.tif]

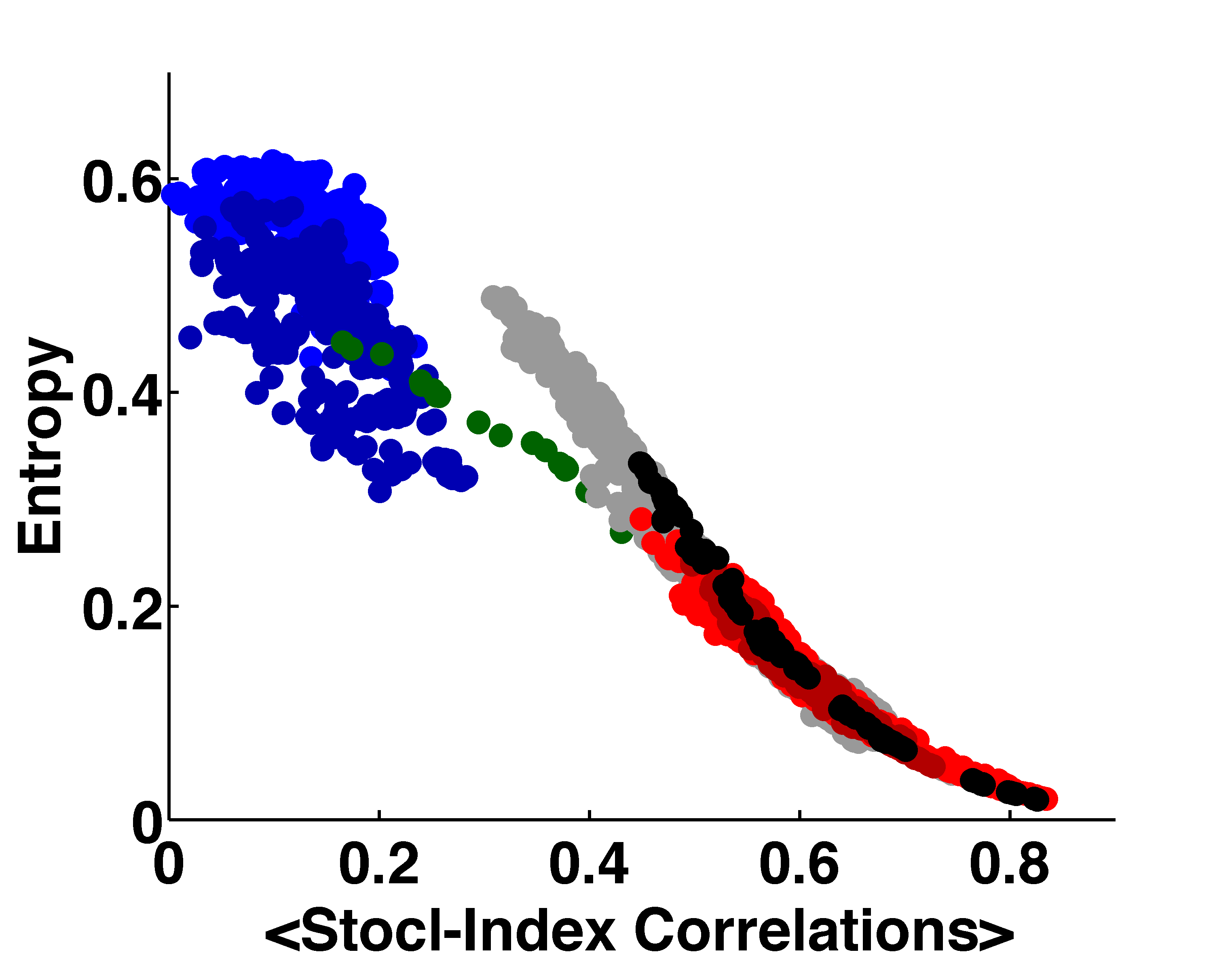

Supplement: Figure S4 — Eigenvalue entropy versus the average stock-index correlation, as function of time, color coded according to Figure 1A. This is presented for the 300 stock subset, as in Figure S1, S2, S3. We first calculate the average value of the entropy and the stock-index correlation over all 4 iterations. (TIF) [file pone.0019378.s004.tif]

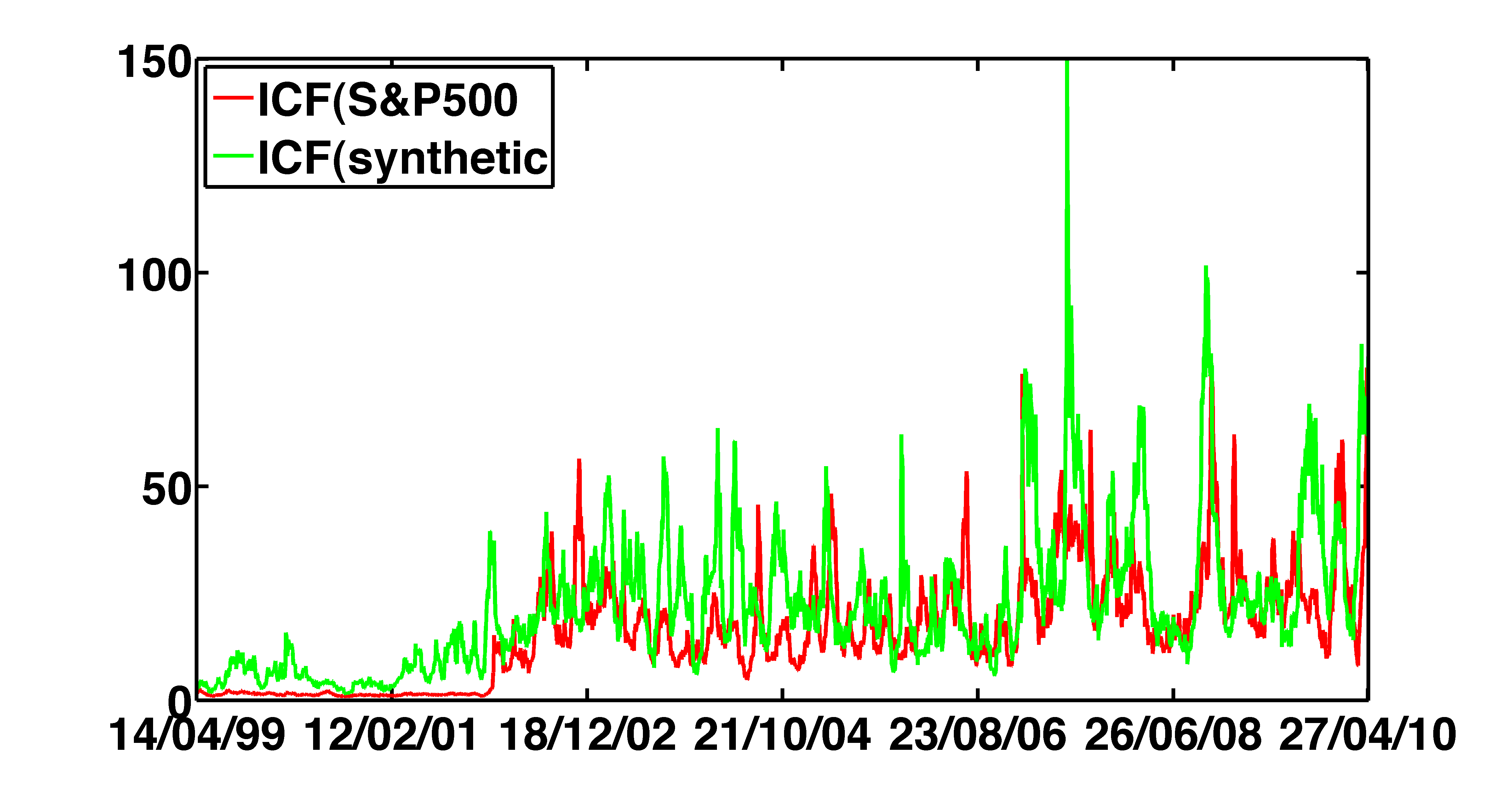

Supplement: Figure S5 — Comparison of the ICF calculated using the S&P500 index (red curve) and the ICF calculated using a synthetic index. The synthetic index was calculated using only the stocks included in the dataset, as a weighted average of these stocks, using their original weights from the S&P500 index. While the ICF calculated using the synthetic index is nosier, the two are qualitatively very similar, with a correlation of 0.65, which is probably strongly affected by the fact that the ICF(synthetic) is much nosier in the pre-2002 period. (TIF) [file pone.0019378.s005.tif]

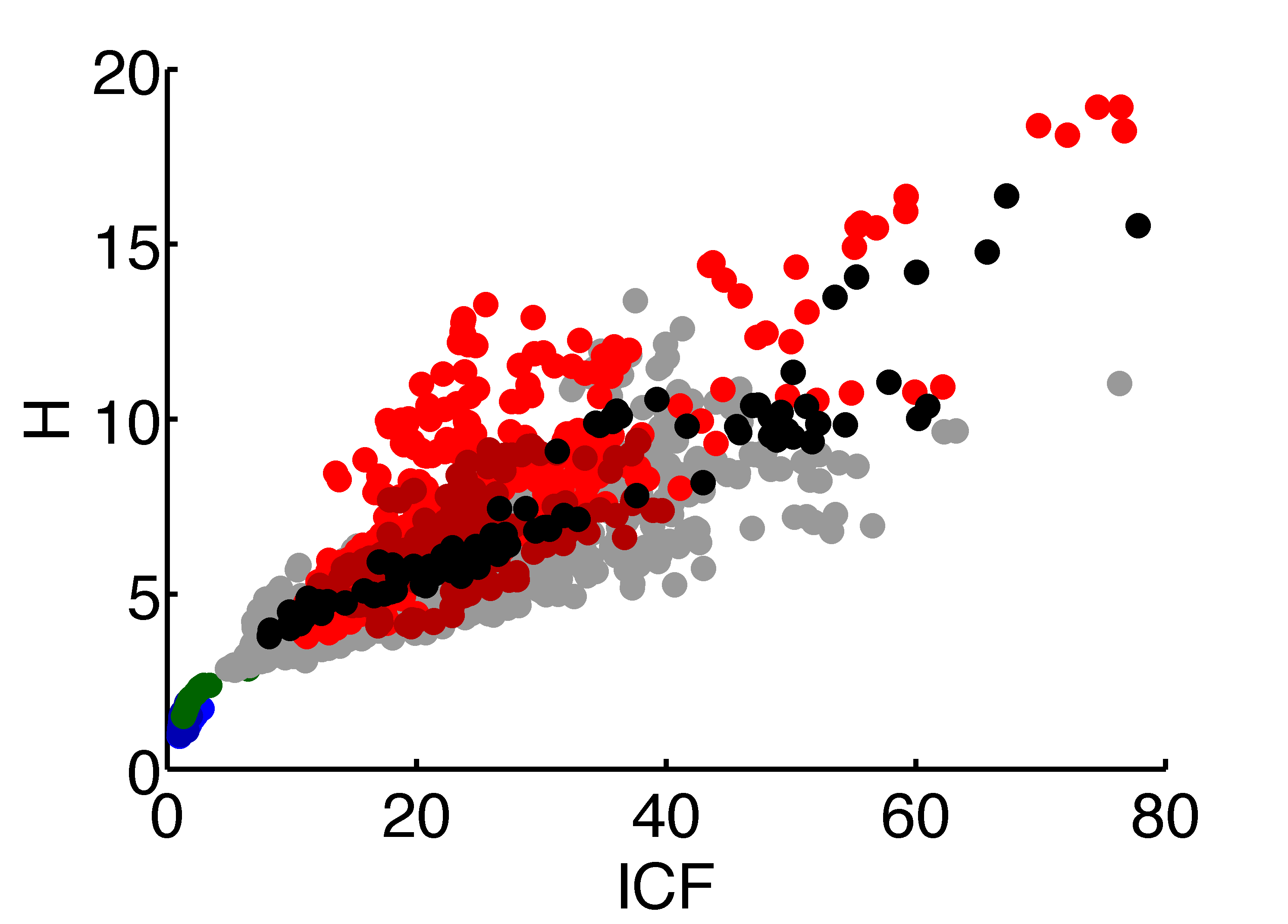

Supplement: Figure S6 — Comparison of the H factor to the ICF, color coded for time according to the code presented in Figure 1A. (TIF) [file pone.0019378.s006.tif]

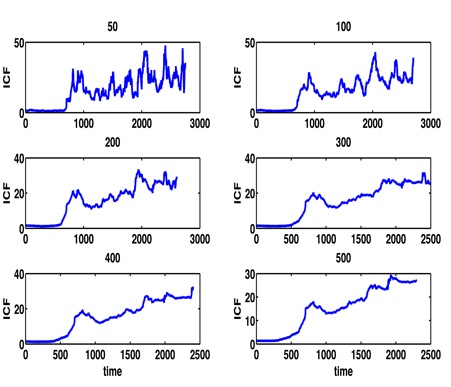

Supplement: Figure S7 — ICF analysis of the S&P500 dataset, using a sliding window of 50, 100, 200, 300, 400, and 500 days. The transition, observed using the 22-day window, is qualitatively observed for all other window sizes, around the same period. (TIFF) [file pone.0019378.s007.tiff]

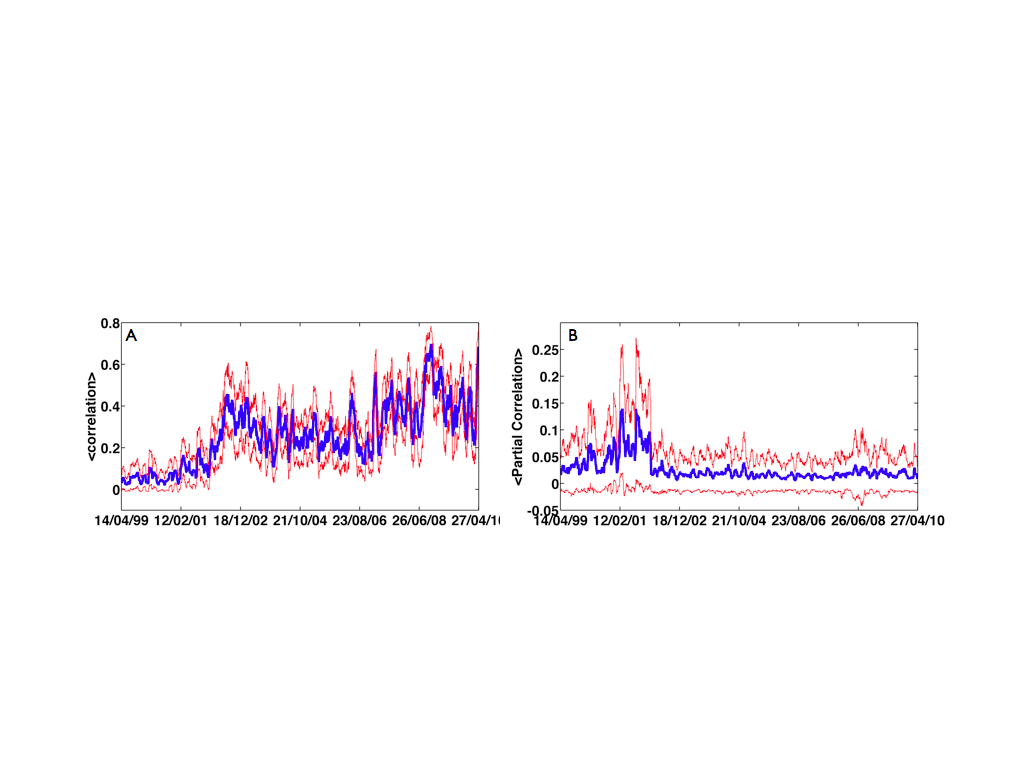

Supplement: Figure S8 — The average stock correlation (left) and average stock partial correlation (right), as resented in Figure 1 A and B respectively, with the addition of error lines. The error lines were estimated using the standard deviation for each parameter separately, marked by a dotted red line. (TIFF) [file pone.0019378.s008.tiff]

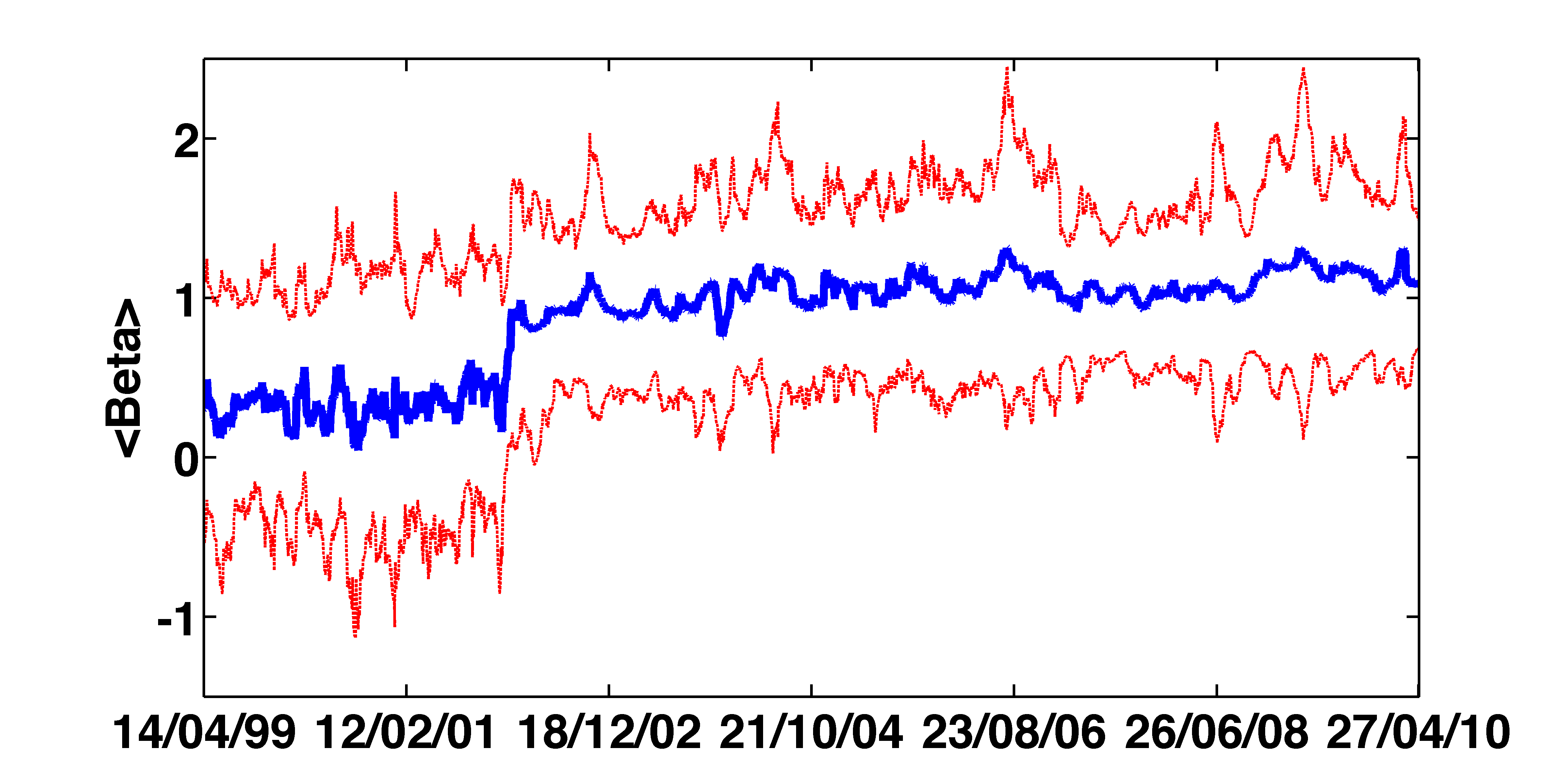

Supplement: Figure S9 — The average Beta coefficient, as presented in Figure 4, with the addition of error, estimated using the standard deviation, marked with a dotted red line. (TIF) [file pone.0019378.s009.tif]

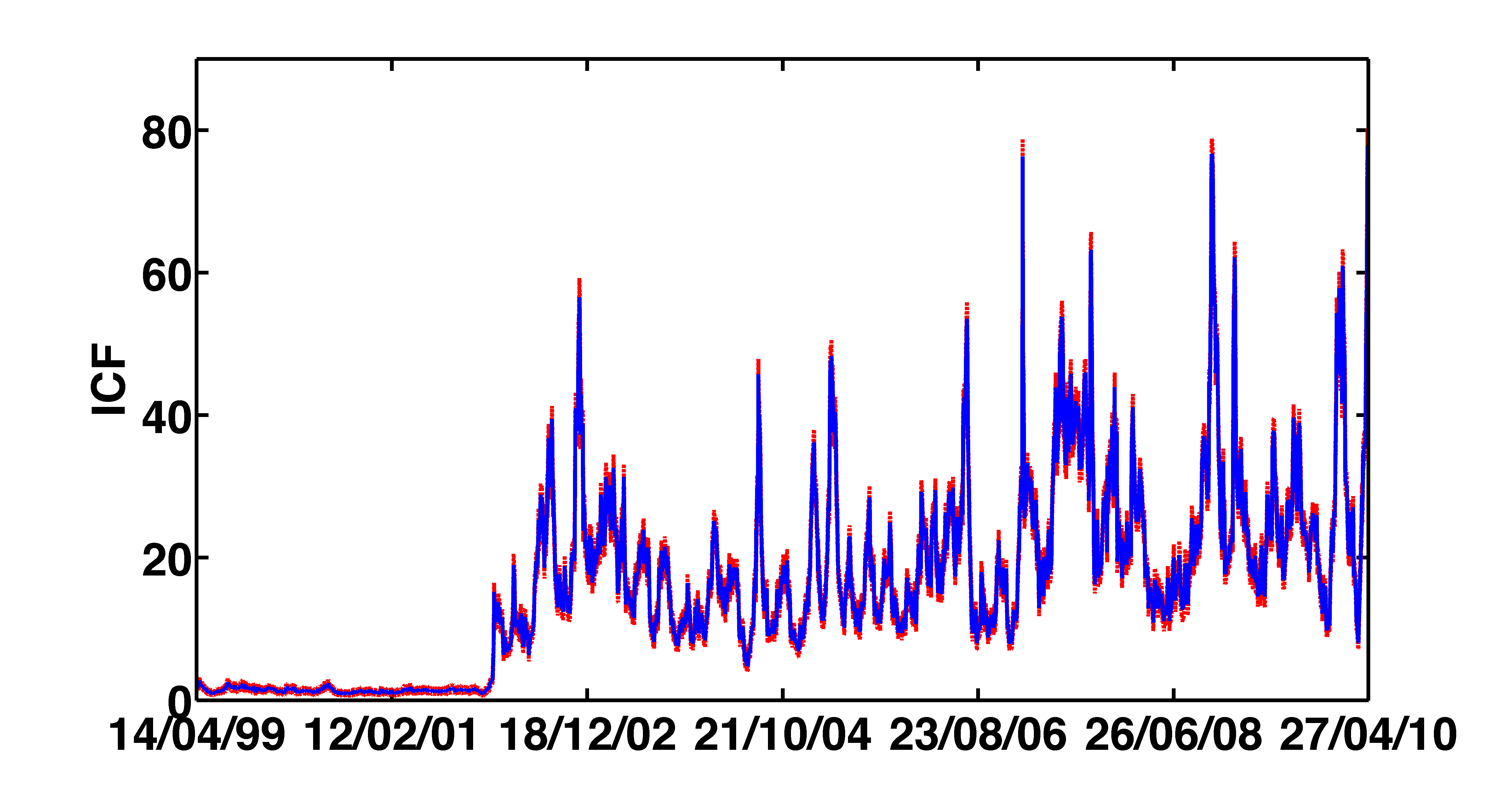

Supplement: Figure S10 — The value of the ICF as a function of time, with the addition of error markers, estimated by the standard deviations of the average correlation and average partial correlation, and the functional relation between them. The error boundaries are marked by a dotted red line. (TIF) [file pone.0019378.s010.tif]
