# Supplementary material for: Index Cohesive Force Analysis Reveals That the US Market Became Prone to Systemic Collapses Since 2002
Source: PLoS One. 2011 Apr 27;6(4):e19378. doi: 10.1371/journal.pone.0019378 (PMC3083438; doi:10.1371/journal.pone.0019378)
Supplement: Text S1 — (DOC) [file pone.0019378.s011.doc]

Index cohesive force analysis reveals that the US market became prone to systemic collapses since 2002

D.Y. Kenett^1^, Y. Shapira^1^, A. Madi^2^, S. Bransburg-Zabary^2^, G. Gur-Gershgoren^3,4^, and E. Ben-Jacob^1,#^

^1^ School of Physics and Astronomy, Tel-Aviv University, Tel-Aviv, Israel

^2^ Faculty of Medicine, Tel-Aviv University, Tel-Aviv, Israel

^3^ School of Business and Management, Ben Gurion University, Beer Sheva, Israel

^4^ Department of Economic Research, Israel Securities Authority,Jerusalem, Israel

^(#)^ Corresponding author email [eshelbj@gmail.com](mailto:eshelbj@gmail.com)

Text S1: Data Processing and validation

The dataset analyzed in this study is of stocks belonging to the S&P500 index. The list of stocks belonging to the index as of April 2010 was used to choose stocks for this study. Once the stocks were selected, their historical data was downloaded from the Yahoo! website ([www.finance.yahoo.com](http://www.finance.yahoo.com/)).

For each stock, we made use of the daily adjusted closing price, $P_{i}\left(t\right)$, to calculate the time series of daily return, calculated as

$r_{i}\left(t\right) = \log\left[P_{i}\left(t\right)\right] - \log\left[P_{i}\left(t-1\right)\right]$ (S-1)

Finally, we choose out of the 500 stocks, only stocks that had data for the entire time period (April 1999 – April 2010). This left us with 418 stocks. In Table S1 we present the breakup of the remaining stocks into the different financial sectors.

As a further validation of the results obtained using the dataset containing 418 stocks, we repeated the ICF analysis for the period of 2010, with all of the 500 S&P500 stocks. Then, as we presented in Figure 2, we compare the ICF to the average stock-index correlations. We use a color code to present the chronological time progression, from dark blue at the beginning of 2010. to dark red at April 2010. We compare the results obtained for the 500 stocks with those obtained for the 418 stocks. As can be seen in Figure S1, the results are of high similarity.

Table S1: Number of stocks, out of the 418 stocks in the dataset, belonging to the different financial sectors.

|  | Sector | # of stocks |
| --- | --- | --- |
| 1 | Consumer Discretionary | 67 |
| 2 | Consumer Staples | 34 |
| 3 | Energy | 36 |
| 4 | Financials | 61 |
| 5 | Health Care | 43 |
| 6 | Industrials | 53 |
| 7 | Information Technology | 61 |
| 8 | Materials | 26 |
| 9 | Telecommunications Services | 7 |
| 10 | Utilities | 32 |

To further validate the dataset, which contained 418 out of the 500 S&P500 stocks, we randomly selected a set of 300 stocks out of our dataset, and repeated the calculations of the correlations, partial correlations, entropy and ICF. We repeated this sub-set selection 4 iterations. In Figure S2 we present the dynamics of the ICF, for each iteration of the random selection process. Each curve is marked using a different color. Next, in Figure S3 we repeat the analysis presented in Figure 8. We calculate the average value of the average raw correlation, average stock-index correlation, and average partial correlation, over all 100 iterations for each time window. Finally, in Figure S4 we compare the entropy to the average stock-index correlation, after once again first averaging over all 4 iterations.

As a final validation test, we constructed a synthetic index out of the stocks belonging to our dataset. For each day, we calculated the weighted average of stocks contained in the dataset, using their original weights in the S&P500 index. We then re-analyzed the data, and calculated the ICF using the synthetic index. In Figure S5 we present the ICF as a function of time calculated using the S&P500 index (red) and the synthetic index (green). While the ICF is noisier when calculated using the synthetic index, especially in the pre-2002 period, it is still qualitatively very similar to the ICF calculated using the S&P500 index. The Pearson correlation between the two is 0.65, which is probably higher for the post-2002 period.

Finally, we propose one possible generalization of the ICF. This is achieved by a normalization of the ICF by the STD’s of the correlations and partial correlations, defined as

$H= \frac{\overline{\left\langle C\left(i,j\right)\right\rangle_{\tau}}}{\overline{\left\langle\rho\left(\left.i,j \right| m\right)\right\rangle_{\tau}}}\cdot\left(\frac{\sigma\left(\left\langle C\left(i,j\right)\right\rangle_{t}\right)}{\sigma\left(\left\langle\rho\left(\left.i,j \right| m\right)\right\rangle\right)}\right)^{-1}=ICF\left(\tau\right)\cdot\left(\frac{\sigma\left(\left\langle C\left(i,j\right)\right\rangle_{t}\right)}{\sigma\left(\left\langle\rho\left(\left.i,j \right| m\right)\right\rangle\right)}\right)^{-1}$ (S-2)

According to this definition, H is the Herd factor, which is the ICF normalized by the volatility, expressed by the ratio of the STD of the correlations and partial correlations. We propose this factor as a measure to quantify the herding effect in the markets. In Figure S6 we present a comparison of the H factor to the ICF, color-coded by time according to Figure 1A. A full description of the H factor will be presented elsewhere.
