# Supplementary material for: Index Cohesive Force Analysis Reveals That the US Market Became Prone to Systemic Collapses Since 2002
Source: PLoS One. 2011 Apr 27;6(4):e19378. doi: 10.1371/journal.pone.0019378 (PMC3083438; doi:10.1371/journal.pone.0019378)
Supplement: Text S2 — (DOC) [file pone.0019378.s012.doc]

Index cohesive force analysis reveals that the US market became prone to systemic collapses since 2002

D.Y. Kenett^1^, Y. Shapira^1^, A. Madi^2^, S. Bransburg-Zabary^2^, G. Gur-Gershgoren^3,4^, and E. Ben-Jacob^1,#^

^1^ School of Physics and Astronomy, Tel-Aviv University, Tel-Aviv, Israel

^2^ Faculty of Medicine, Tel-Aviv University, Tel-Aviv, Israel

^3^ School of Business and Management, Ben Gurion University, Beer Sheva, Israel

^4^ Department of Economic Research, Israel Securities Authority, Jerusalem, Israel

^(#)^ Corresponding author email [eshelbj@gmail.com](mailto:eshelbj@gmail.com)

Text S2: ICF analysis with windows of different sizes

In this study we use a short, 22-day time window. To validate the results obtained using this window, we repeat the ICF calculation, using windows of different sizes. We used a 50, 100, 200, 300, 400, and 500 day window. In Figure S7 we present the ICF as function of window size. It is possible to observe that the transition in market structure, observed for the 22-day window, is observed for all other window sizes. However, as the size of the window increases, the transition
