# Supplementary material for: Index Cohesive Force Analysis Reveals That the US Market Became Prone to Systemic Collapses Since 2002
Source: PLoS One. 2011 Apr 27;6(4):e19378. doi: 10.1371/journal.pone.0019378 (PMC3083438; doi:10.1371/journal.pone.0019378)
Supplement: Text S3 — (DOC) [file pone.0019378.s013.doc]

Index cohesive force analysis reveals that the US market became prone to systemic collapses since 2002

D.Y. Kenett^1^, Y. Shapira^1^, A. Madi^2^, S. Bransburg-Zabary^2^, G. Gur-Gershgoren^3,4^, and E. Ben-Jacob^1,#^

^1^ School of Physics and Astronomy, Tel-Aviv University, Tel-Aviv, Israel

^2^ Faculty of Medicine, Tel-Aviv University, Tel-Aviv, Israel

^3^ School of Business and Management, Ben Gurion University, Beer Sheva, Israel

^4^ Department of Economic Research, Israel Securities Authority, Jerusalem, Israel

^(#)^ Corresponding author email [eshelbj@gmail.com](mailto:eshelbj@gmail.com)

Text S3: Error estimation of correlation analysis

In this appendix we present the key results discussed in the main text, with the addition of error estimation for each parameter. To estimate the error, we make use of the Standard deviation (STD).

In Figure S8 we present the average stock correlation (left) and the average stock partial correlation (right), as presented in Figure 1 of the main text, with error bars which indicate the $\pm1SD$. In Figure S9 we present the average $\beta$coefficient, as presented in Figure 4 of the main text, with the addition of the $\pm1SD$ error bars.

Finally, in Figure S10 we present the ICF for the entire period, with the addition of the error bar. To estimate the error for the ICF, we used the error of the average correlation and average partial correlation, and computed the error of the function of the ratio of the two as

$ICF_{error}=\sqrt{\left(\frac{1}{\left\langle\rho\left(i,j\right)\right\rangle}\right)^{2}\cdot STD_{Corr}^{2}+\left(\frac{\left\langle C\left(i,j\right)\right\rangle}{\left\langle\rho\left(i,j\right)\right\rangle^{2}}\right)^{2}\cdot STD_{Parr}^{2}}$ (S-3)
